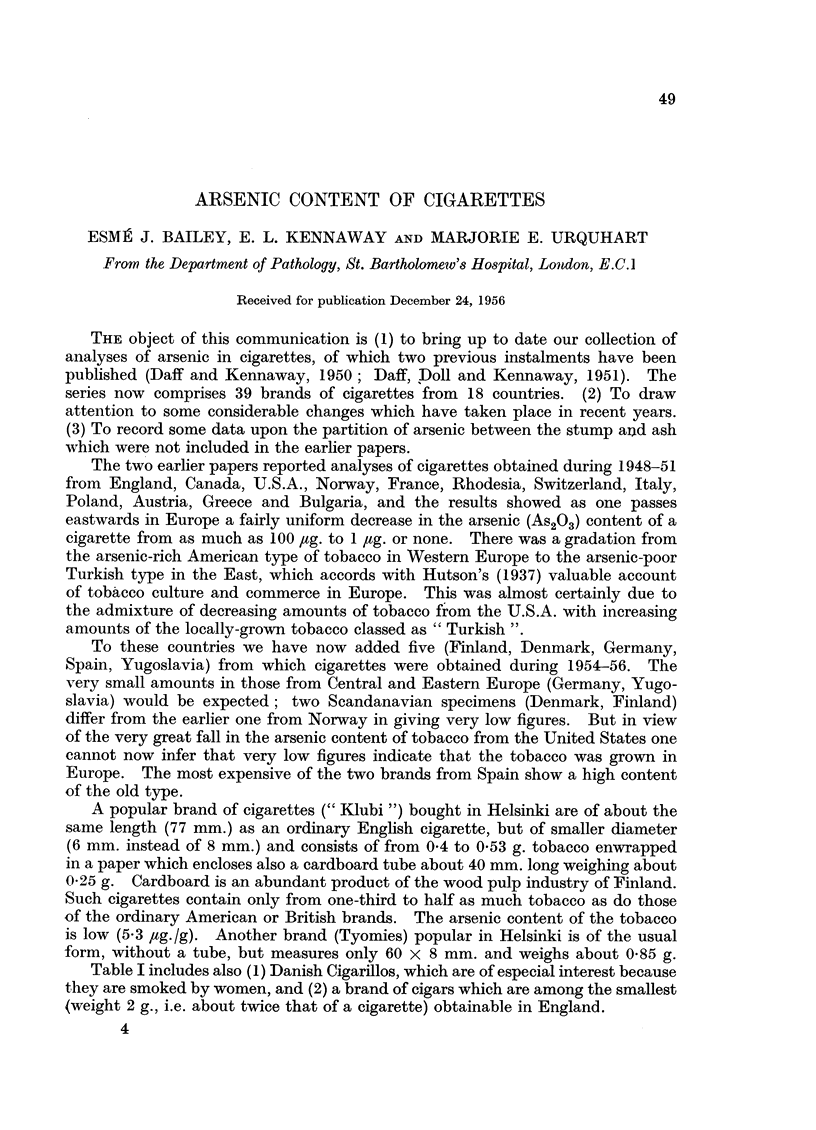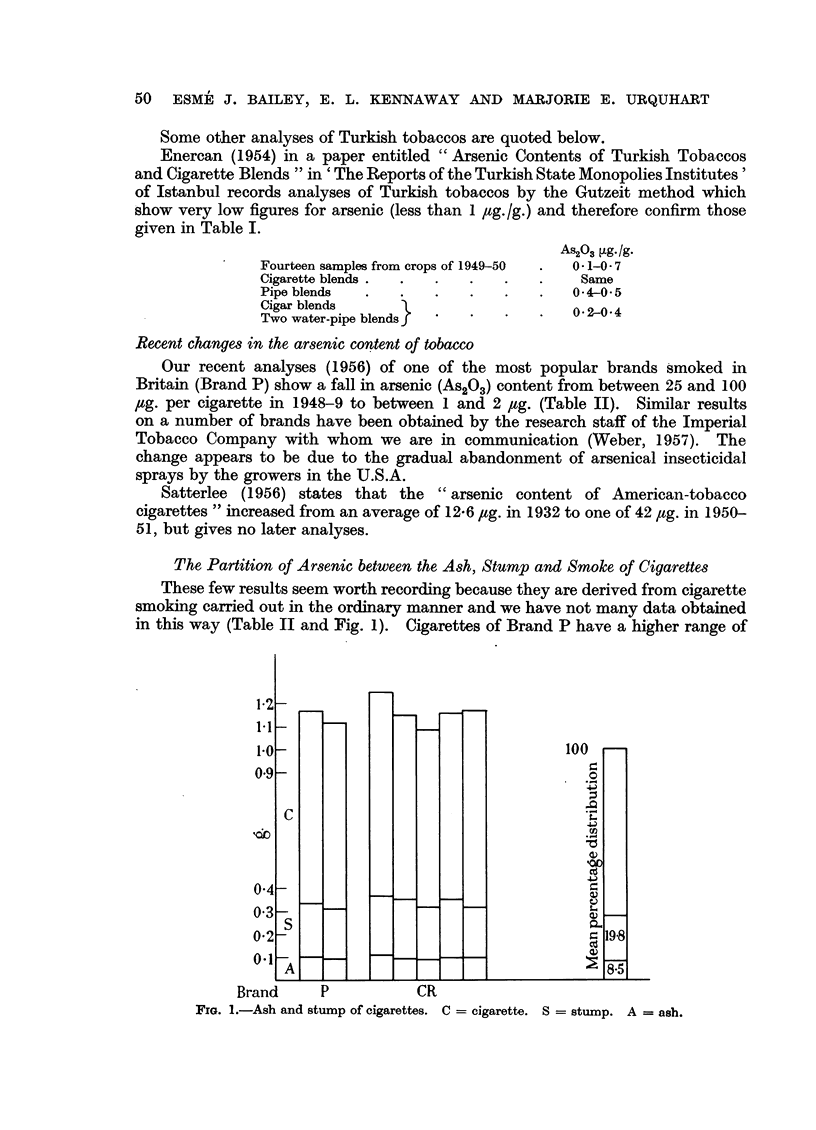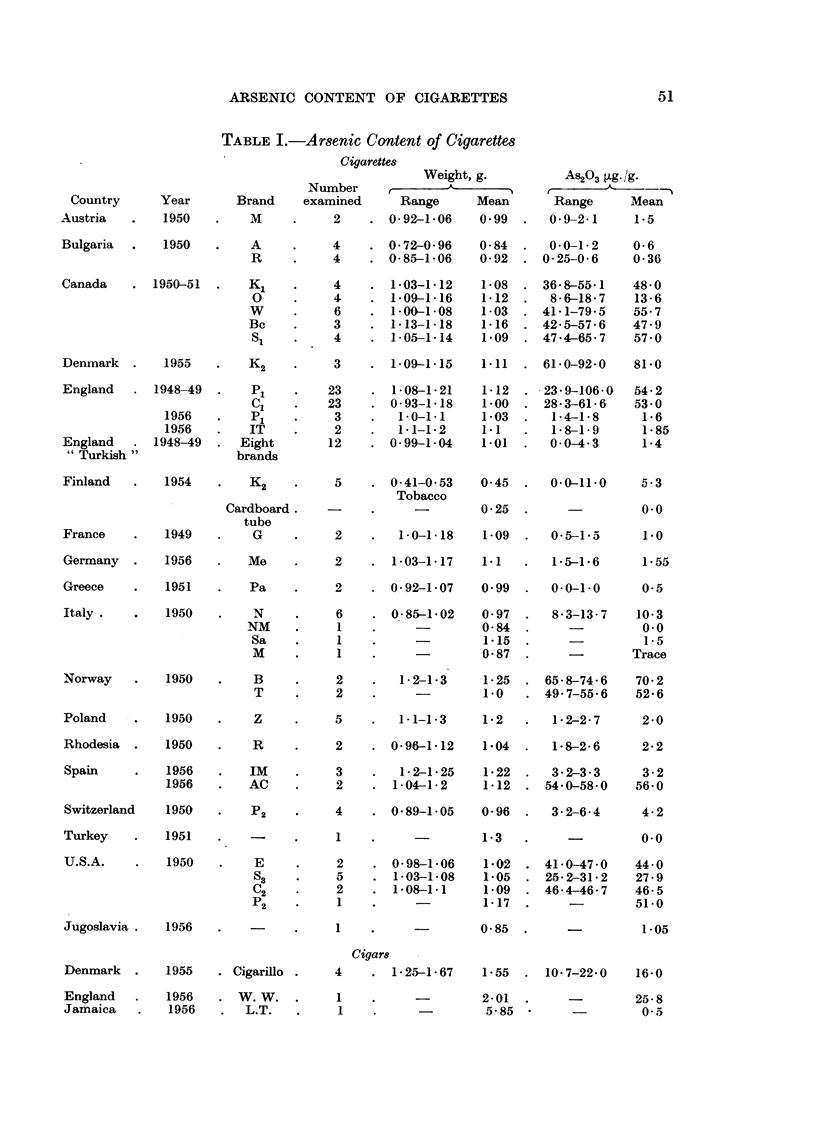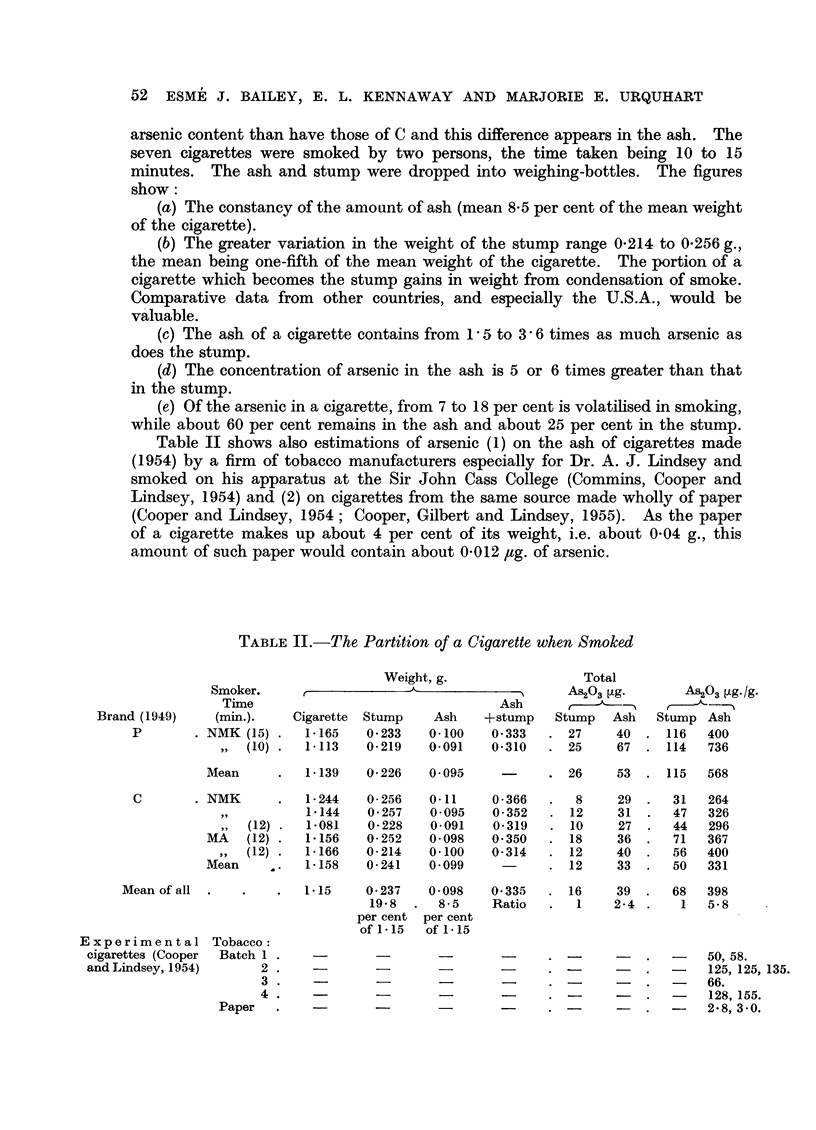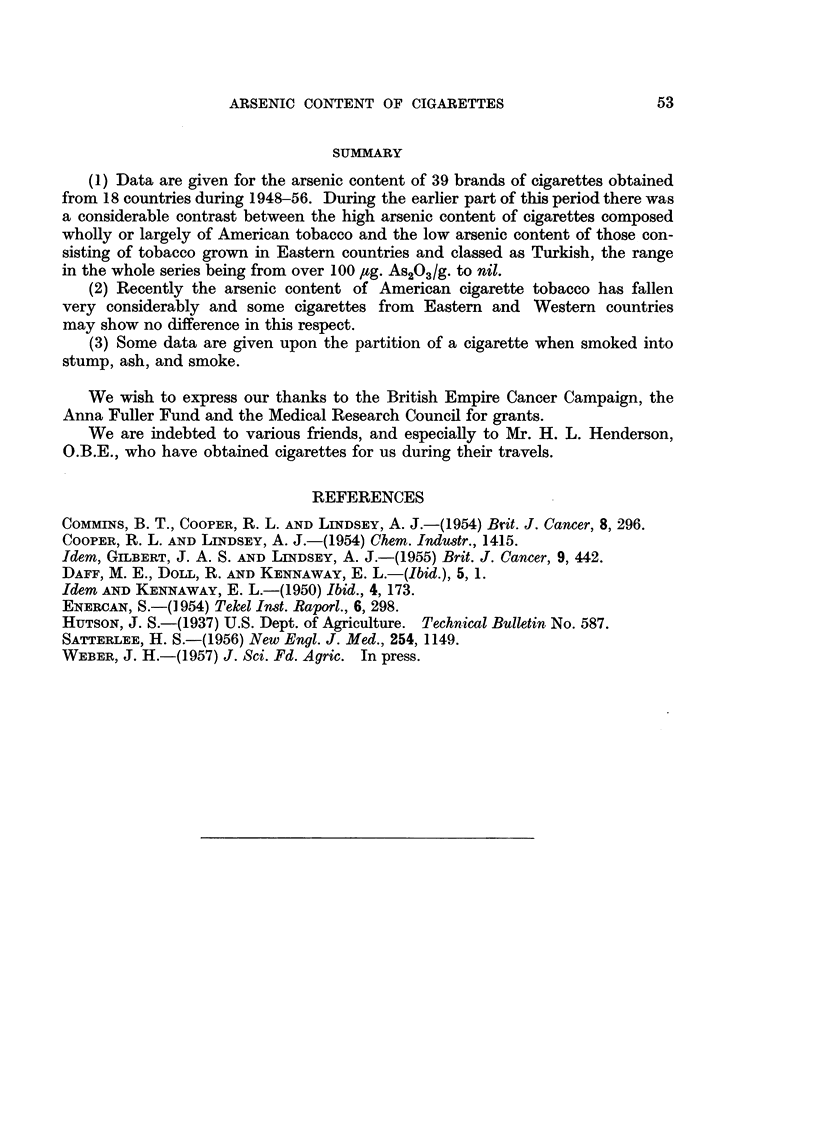# Arsenic Content of Cigarettes

**DOI:** 10.1038/bjc.1957.7

**Published:** 1957-03

**Authors:** Esmé J. Bailey, E. L. Kennaway, Marjorie E. Urquhart


### ARSENIC CONTENT OF CIGARETTES

ESMÉ J. BAILEY, E. L. KENNAWAY AND MARJORIE E. URQUHART

*From the Department of Pathology, St. Bartholomew's Hospital, London, E.C.1*

Received for publication December 24, 1956

THE object of this communication is (1) to bring up to date our collection of analyses of arsenic in cigarettes, of which two previous instalments have been published (Daff and Kennaway, 1950; Daff, Doll and Kennaway, 1951). The series now comprises 39 brands of cigarettes from 18 countries. (2) To draw attention to some considerable changes which have taken place in recent years. (3) To record some data upon the partition of arsenic between the stump and ash which were not included in the earlier papers.

The two earlier papers reported analyses of cigarettes obtained during 1948–51 from England, Canada, U.S.A., Norway, France, Rhodesia, Switzerland, Italy, Poland, Austria, Greece and Bulgaria, and the results showed as one passes eastwards in Europe a fairly uniform decrease in the arsenic ($As_2O_3$) content of a cigarette from as much as 100 $\mu$g. to 1 $\mu$g. or none. There was a gradation from the arsenic-rich American type of tobacco in Western Europe to the arsenic-poor Turkish type in the East, which accords with Hutson's (1937) valuable account of tobacco culture and commerce in Europe. This was almost certainly due to the admixture of decreasing amounts of tobacco from the U.S.A. with increasing amounts of the locally-grown tobacco classed as " Turkish ".

To these countries we have now added five (Finland, Denmark, Germany, Spain, Yugoslavia) from which cigarettes were obtained during 1954–56. The very small amounts in those from Central and Eastern Europe (Germany, Yugoslavia) would be expected; two Scandanavian specimens (Denmark, Finland) differ from the earlier one from Norway in giving very low figures. But in view of the very great fall in the arsenic content of tobacco from the United States one cannot now infer that very low figures indicate that the tobacco was grown in Europe. The most expensive of the two brands from Spain show a high content of the old type.

A popular brand of cigarettes (" Klubi ") bought in Helsinki are of about the same length (77 mm.) as an ordinary English cigarette, but of smaller diameter (6 mm. instead of 8 mm.) and consists of from 0·4 to 0·53 g. tobacco enwrapped in a paper which encloses also a cardboard tube about 40 mm. long weighing about 0·25 g. Cardboard is an abundant product of the wood pulp industry of Finland. Such cigarettes contain only from one-third to half as much tobacco as do those of the ordinary American or British brands. The arsenic content of the tobacco is low (5·3 $\mu$g./g). Another brand (Tyomies) popular in Helsinki is of the usual form, without a tube, but measures only 60 × 8 mm. and weighs about 0·85 g.

Table I includes also (1) Danish Cigarillos, which are of especial interest because they are smoked by women, and (2) a brand of cigars which are among the smallest (weight 2 g., i.e. about twice that of a cigarette) obtainable in England.

Some other analyses of Turkish tobaccos are quoted below.

Enercan (1954) in a paper entitled "Arsenic Contents of Turkish Tobaccos and Cigarette Blends" in 'The Reports of the Turkish State Monopolies Institutes' of Istanbul records analyses of Turkish tobaccos by the Gutzeit method which show very low figures for arsenic (less than 1 $\mu$g./g.) and therefore confirm those given in Table I.

| | $As_2O_3$ $\mu$g./g. |
|---|---|
| Fourteen samples from crops of 1949–50 | 0·1–0·7 |
| Cigarette blends | Same |
| Pipe blends | 0·4–0·5 |
| Cigar blends / Two water-pipe blends | 0·2–0·4 |

*Recent changes in the arsenic content of tobacco*

Our recent analyses (1956) of one of the most popular brands smoked in Britain (Brand P) show a fall in arsenic ($As_2O_3$) content from between 25 and 100 $\mu$g. per cigarette in 1948–9 to between 1 and 2 $\mu$g. (Table II). Similar results on a number of brands have been obtained by the research staff of the Imperial Tobacco Company with whom we are in communication (Weber, 1957). The change appears to be due to the gradual abandonment of arsenical insecticidal sprays by the growers in the U.S.A.

Satterlee (1956) states that the "arsenic content of American-tobacco cigarettes" increased from an average of 12·6 $\mu$g. in 1932 to one of 42 $\mu$g. in 1950–51, but gives no later analyses.

*The Partition of Arsenic between the Ash, Stump and Smoke of Cigarettes*

These few results seem worth recording because they are derived from cigarette smoking carried out in the ordinary manner and we have not many data obtained in this way (Table II and Fig. 1). Cigarettes of Brand P have a higher range of

**Fig. 1.**—Ash and stump of cigarettes. C = cigarette. S = stump. A = ash.

TABLE I.—*Arsenic Content of Cigarettes*

| Country | Year | Brand | Number examined | Weight, g. Range | Weight, g. Mean | $As_2O_3$ µg./g. Range | $As_2O_3$ µg./g. Mean |
|---|---|---|---|---|---|---|---|
| | | | *Cigarettes* | | | | |
| Austria | 1950 | M | 2 | 0·92–1·06 | 0·99 | 0·9–2·1 | 1·5 |
| Bulgaria | 1950 | A | 4 | 0·72–0·96 | 0·84 | 0·0–1·2 | 0·6 |
| | | R | 4 | 0·85–1·06 | 0·92 | 0·25–0·6 | 0·36 |
| Canada | 1950–51 | $K_1$ | 4 | 1·03–1·12 | 1·08 | 36·8–55·1 | 48·0 |
| | | O | 4 | 1·09–1·16 | 1·12 | 8·6–18·7 | 13·6 |
| | | W | 6 | 1·00–1·08 | 1·03 | 41·1–79·5 | 55·7 |
| | | Bc | 3 | 1·13–1·18 | 1·16 | 42·5–57·6 | 47·9 |
| | | $S_1$ | 4 | 1·05–1·14 | 1·09 | 47·4–65·7 | 57·0 |
| Denmark | 1955 | $K_2$ | 3 | 1·09–1·15 | 1·11 | 61·0–92·0 | 81·0 |
| England | 1948–49 | $P_1$ | 23 | 1·08–1·21 | 1·12 | 23·9–106·0 | 54·2 |
| | | $C_1$ | 23 | 0·93–1·18 | 1·00 | 28·3–61·6 | 53·0 |
| | 1956 | $P_1$ | 3 | 1·0–1·1 | 1·03 | 1·4–1·8 | 1·6 |
| | 1956 | IT | 2 | 1·1–1·2 | 1·1 | 1·8–1·9 | 1·85 |
| England "Turkish" | 1948–49 | Eight brands | 12 | 0·99–1·04 | 1·01 | 0·0–4·3 | 1·4 |
| Finland | 1954 | $K_2$ | 5 | 0·41–0·53 | 0·45 | 0·0–11·0 | 5·3 |
| | | Cardboard tube | — | — Tobacco | 0·25 | — | 0·0 |
| France | 1949 | G | 2 | 1·0–1·18 | 1·09 | 0·5–1·5 | 1·0 |
| Germany | 1956 | Me | 2 | 1·03–1·17 | 1·1 | 1·5–1·6 | 1·55 |
| Greece | 1951 | Pa | 2 | 0·92–1·07 | 0·99 | 0·0–1·0 | 0·5 |
| Italy | 1950 | N | 6 | 0·85–1·02 | 0·97 | 8·3–13·7 | 10·3 |
| | | NM | 1 | — | 0·84 | — | 0·0 |
| | | Sa | 1 | — | 1·15 | — | 1·5 |
| | | M | 1 | — | 0·87 | — | Trace |
| Norway | 1950 | B | 2 | 1·2–1·3 | 1·25 | 65·8–74·6 | 70·2 |
| | | T | 2 | — | 1·0 | 49·7–55·6 | 52·6 |
| Poland | 1950 | Z | 5 | 1·1–1·3 | 1·2 | 1·2–2·7 | 2·0 |
| Rhodesia | 1950 | R | 2 | 0·96–1·12 | 1·04 | 1·8–2·6 | 2·2 |
| Spain | 1956 | IM | 3 | 1·2–1·25 | 1·22 | 3·2–3·3 | 3·2 |
| | 1956 | AC | 2 | 1·04–1·2 | 1·12 | 54·0–58·0 | 56·0 |
| Switzerland | 1950 | $P_2$ | 4 | 0·89–1·05 | 0·96 | 3·2–6·4 | 4·2 |
| Turkey | 1951 | — | 1 | — | 1·3 | — | 0·0 |
| U.S.A. | 1950 | E | 2 | 0·98–1·06 | 1·02 | 41·0–47·0 | 44·0 |
| | | $S_3$ | 5 | 1·03–1·08 | 1·05 | 25·2–31·2 | 27·9 |
| | | $C_2$ | 2 | 1·08–1·1 | 1·09 | 46·4–46·7 | 46·5 |
| | | $P_2$ | 1 | — | 1·17 | — | 51·0 |
| Jugoslavia | 1956 | — | 1 | — | 0·85 | — | 1·05 |
| | | | *Cigars* | | | | |
| Denmark | 1955 | Cigarillo | 4 | 1·25–1·67 | 1·55 | 10·7–22·0 | 16·0 |
| England | 1956 | W. W. | 1 | — | 2·01 | — | 25·8 |
| Jamaica | 1956 | L.T. | 1 | — | 5·85 | — | 0·5 |

arsenic content than have those of C and this difference appears in the ash. The seven cigarettes were smoked by two persons, the time taken being 10 to 15 minutes. The ash and stump were dropped into weighing-bottles. The figures show :

(*a*) The constancy of the amount of ash (mean 8·5 per cent of the mean weight of the cigarette).

(*b*) The greater variation in the weight of the stump range 0·214 to 0·256 g., the mean being one-fifth of the mean weight of the cigarette. The portion of a cigarette which becomes the stump gains in weight from condensation of smoke. Comparative data from other countries, and especially the U.S.A., would be valuable.

(*c*) The ash of a cigarette contains from 1·5 to 3·6 times as much arsenic as does the stump.

(*d*) The concentration of arsenic in the ash is 5 or 6 times greater than that in the stump.

(*e*) Of the arsenic in a cigarette, from 7 to 18 per cent is volatilised in smoking, while about 60 per cent remains in the ash and about 25 per cent in the stump.

Table II shows also estimations of arsenic (1) on the ash of cigarettes made (1954) by a firm of tobacco manufacturers especially for Dr. A. J. Lindsey and smoked on his apparatus at the Sir John Cass College (Commins, Cooper and Lindsey, 1954) and (2) on cigarettes from the same source made wholly of paper (Cooper and Lindsey, 1954 ; Cooper, Gilbert and Lindsey, 1955). As the paper of a cigarette makes up about 4 per cent of its weight, i.e. about 0·04 g., this amount of such paper would contain about 0·012 $\mu$g. of arsenic.

TABLE II.—*The Partition of a Cigarette when Smoked*

| Brand (1949) | Smoker. Time (min.). | Weight, g. Cigarette | Stump | Ash | Ash +stump | Total $As_2O_3$ $\mu$g. Stump | Ash | $As_2O_3$ $\mu$g./g. Stump | Ash |
|---|---|---|---|---|---|---|---|---|---|
| P | NMK (15) | 1·165 | 0·233 | 0·100 | 0·333 | 27 | 40 | 116 | 400 |
| | „ (10) | 1·113 | 0·219 | 0·091 | 0·310 | 25 | 67 | 114 | 736 |
| | Mean | 1·139 | 0·226 | 0·095 | — | 26 | 53 | 115 | 568 |
| C | NMK | 1·244 | 0·256 | 0·11 | 0·366 | 8 | 29 | 31 | 264 |
| | „ | 1·144 | 0·257 | 0·095 | 0·352 | 12 | 31 | 47 | 326 |
| | „ (12) | 1·081 | 0·228 | 0·091 | 0·319 | 10 | 27 | 44 | 296 |
| | MA (12) | 1·156 | 0·252 | 0·098 | 0·350 | 18 | 36 | 71 | 367 |
| | „ (12) | 1·166 | 0·214 | 0·100 | 0·314 | 12 | 40 | 56 | 400 |
| | Mean | 1·158 | 0·241 | 0·099 | — | 12 | 33 | 50 | 331 |
| Mean of all | | 1·15 | 0·237 | 0·098 | 0·335 | 16 | 39 | 68 | 398 |
| | | | 19·8 per cent of 1·15 | 8·5 per cent of 1·15 | Ratio | 1 | 2·4 | 1 | 5·8 |
| Experimental cigarettes (Cooper and Lindsey, 1954) | Tobacco : Batch 1 | — | — | — | — | — | — | — | 50, 58. |
| | 2 | — | — | — | — | — | — | — | 125, 125, 135. |
| | 3 | — | — | — | — | — | — | — | 66. |
| | 4 | — | — | — | — | — | — | — | 128, 155. |
| | Paper | — | — | — | — | — | — | — | 2·8, 3·0. |

SUMMARY

(1) Data are given for the arsenic content of 39 brands of cigarettes obtained from 18 countries during 1948–56. During the earlier part of this period there was a considerable contrast between the high arsenic content of cigarettes composed wholly or largely of American tobacco and the low arsenic content of those consisting of tobacco grown in Eastern countries and classed as Turkish, the range in the whole series being from over 100 $\mu$g. $As_2O_3$/g. to *nil.*

(2) Recently the arsenic content of American cigarette tobacco has fallen very considerably and some cigarettes from Eastern and Western countries may show no difference in this respect.

(3) Some data are given upon the partition of a cigarette when smoked into stump, ash, and smoke.

We wish to express our thanks to the British Empire Cancer Campaign, the Anna Fuller Fund and the Medical Research Council for grants.

We are indebted to various friends, and especially to Mr. H. L. Henderson, O.B.E., who have obtained cigarettes for us during their travels.

### REFERENCES

Commins, B. T., Cooper, R. L. and Lindsey, A. J.—(1954) *Brit. J. Cancer*, **8**, 296.
Cooper, R. L. and Lindsey, A. J.—(1954) *Chem. Industr.*, 1415.
*Idem*, Gilbert, J. A. S. and Lindsey, A. J.—(1955) *Brit. J. Cancer*, **9**, 442.
Daff, M. E., Doll, R. and Kennaway, E. L.—(*Ibid.*), **5**, 1.
*Idem* and Kennaway, E. L.—(1950) *Ibid.*, **4**, 173.
Enercan, S.—(1954) *Tekel Inst. Raporl.*, **6**, 298.
Hutson, J. S.—(1937) U.S. Dept. of Agriculture. *Technical Bulletin* No. 587.
Satterlee, H. S.—(1956) *New Engl. J. Med.*, **254**, 1149.
Weber, J. H.—(1957) *J. Sci. Fd. Agric.* In press.